# Epidemiokinetic Tools to Monitor Lockdown Efficacy and Estimate the Duration Adequate to Control SARS-CoV-2 Spread

**DOI:** 10.1007/s44197-021-00007-3

**Published:** 2021-09-20

**Authors:** Bruno Mégarbane, Fanchon Bourasset, Jean-Michel Scherrmann

**Affiliations:** 1 Department of Medical and Toxicological Critical Care, Lariboisière Hospital, Federation of Toxicology APHP, University of Paris, INSERM UMRS-1144, Paris, France; 2 Laboratory of Integrative Research in Neurosciences and Cognitive Psychology, Bourgogne Franche-Comté University, Besancon, France; 3 Laboratory of Pharmacokinetics, Faculty of Pharmacy, University of Paris, INSERM UMRS-1144, Paris, France

**Keywords:** COVID-19, Lockdown, Duration, Epidemic half-life, Prediction

## Abstract

Various key performance indicators (KPIs) are communicated daily to the public by health authorities since the COVID-19 pandemic has started. “Upstream” KPIs mainly include the incidence of detected Sars-CoV-2-positive cases in the population, and “downstream” KPIs include daily hospitalizations, intensive care unit admissions and fatalities. Whereas “downstream” KPIs are essential to evaluate and adapt hospital organization, “upstream” KPIs are the most appropriate to decide on the strength of restrictions such as lockdown set up and evaluate their effectiveness. Here, we suggested tools derived from pharmacokinetic calculations to improve understanding the epidemic progression. From the time course of the number of new cases of SARS-coV-2 infection in the population, it is possible to calculate the infection rate constant using a simple linear regression and determine its corresponding half-life. This epidemic regression half-life is helpful to measure the potential benefits of restriction measures and to estimate the adequate duration of lockdown if implemented by policymakers in relation to the decided public health objectives. In France, during the first lockdown, we reported an epidemic half-life of 10 days. Our tools allow clearly acknowledging that the zero-COVID target is difficult to reach after a period of lockdown as seven half-lives are required to clear 99.2% of the epidemic and more than 10 half-lives to almost reach the objective of eliminating 100% of the contaminations.

## SARS-CoV-2 Pandemic

The world is facing an unimaginable pandemic attributed to severe acute respiratory syndrome coronavirus 2 (SARS-CoV-2) with > 190 million infected persons and ~ 4.1 million deaths by mid-July 2021 [1]. In the absence of consistently effective anti-SARS-CoV-2 treatments, non-pharmaceutical interventions have been applied by policymakers worldwide to limit the virus spreading and protect the populations [2]. Measures and schedules varied from one country to another with strategies taking into account the different national situations and lifestyles. To gain acceptability by populations, decisions restricting social interactions and economic life relied on easily understandable accurate markers.

Different key performance indicators (KPIs) are communicated daily to the public by health authorities in almost all countries. However, KPI diversity may be confusing with divergent interpretations at the specialist, media and public levels. SARS-CoV-2 epidemic KPIs can be divided into two groups with different temporality. Early KPIs, which we would like to call “upstream”, include quantitative data on viral load measured in wastewater (if available) and incidence of detected SARS-CoV-2-positive cases in the population. Late KPIs, which we would like to call “downstream”, include daily consultations by community doctors, hospitalizations, intensive care unit admissions and fatalities.

## Lockdown to Control the Epidemic

In almost all European countries (except notably Sweden), the SARS-CoV-2 epidemic was responsible for two waves in March–April and October–November 2020. In both periods, governments implemented nationwide lockdowns due to the alarming pressure on hospitals and fearing an increase in deaths. The first strict lockdowns included stay-at-home order, workplace restriction, and school and venue closures. The second lockdowns, less strict in the majority of countries, variably kept schools open and/or maintained a part of the economic activities, consequently tolerating reduced social interaction and circulation of preserved sector workers. While first lockdowns reduced SARS-CoV-2 spreading efficiently to a very low level (e.g., < 500 contaminations/day in France), second lockdowns maintained contaminations at insufficiently lowered levels or plateaus (e.g., ~ 10,000 contaminations/day in France), rapidly followed by progressively increasing trends that indicated the requirement of third lockdowns to control the epidemic. Catastrophic projections and apprehension resulted from new SARS-CoV-2-variant emergence [3], especially the UK variant of concern B.1.1.7, reported to be more infectious and more likely to be linked to increased death rate [4]. Variable prophylactic responses were given ranging from mild restrictions (Spain) to regional/nationwide curfews (France) or strict lockdowns (Germany and Italy).

## Which Key Performance Indicator Should be Used to Monitor Lockdown Effectiveness?

At each stage of the decision process, KPIs, sometimes based on mass testing, were used to interpret the adequacy and efficacy of the strategy [5]. To this end, while the “downstream” KPIs have an inescapable value to evaluate and adapt hospital organization, the “upstream” KPIs seem the most appropriate to decide on the strength of restrictions and evaluate their effectiveness. Monitoring the evolution of daily incidence of SARS-CoV-2 contaminations can be analyzed by the absolute number itself and by the rate at which it evolves. Interestingly, we used the infection incidence rate to analyze lockdown-related efficacy during the March–April 2020 wave [6] and curfew-related efficacy during October 2020–April 2021 period [7]. Although available with delay and highly dependent on rainfalls and population density, data reporting the dynamics of SARS-CoV-2 genome quantification in wastewater may be of great help, as shown during the lockdown in the Paris area [8].

## The Epidemiokinetic Tools

Our suggested tools use calculations derived from pharmacokinetic principles. Our approach is based on a simpler model with fewer parameters than the traditional compartmental susceptible-infected-recovered (SIR) and other derived more stochastic models [9], which nevertheless share similarities with the compartmental pharmacokinetic models. We developed a closed three-compartmental model that considers an input function representing the epidemic progression and an output function representing its regression. We measure the rate and the corresponding half-life issued from the exponential process at which the epidemic progresses rather than the extent of its progression. Our method relies on the predictive power of the input function as surveillance tool. We thus named the parameters obtained using our kinetic calculations “epidemiokinetic tools”.

By contrast to other upstream KPIs such as SARS-CoV-2 genome quantification in wastewater, KPIs based on the epidemiokinetic tools can be obtained using an affordable and universally suitable method. Access to the half-life indicator adds the time dimension as the duration of the exponential process is closely determined by a defined number of half-lives (50% of the kinetic extent process occurs during one half-life). Based on the calculated half-life, epidemic progression or regression duration can be easily estimated. Although underestimated due to limited testing, the number of SARS-CoV-2-infected persons represents an actual sample allowing the confident quantification of the epidemic spread irrespective of the country policy. Our approach supports the contribution of simple mean-field models to evaluate epidemic kinetics and lockdown-attributed effects. However, SARS-CoV-2 genome quantification in wastewater, if available, can probably better predict the epidemic progression or regression on a very local scale.

## The Epidemic Regression Half-Life

We would like to encourage the use as KPI of the “rate” parameter determined from the time-course of cumulative positive cases. Our approach is part of an inter-disciplinary approach based on the analogy of exponential processes between drug elimination in pharmacology and lockdown-related epidemic regression. From the time-course of the number of new cases of SARS-CoV-2 infection in the population, daily determined by the health authorities, it is possible, in each phase of the curve, to calculate the infection rate constant (β) using a simple linear regression and determine its corresponding half-life (t_1/2β_ = ln2/β).

Calculating a regression speed (rate) of an exponential process provides a useful parameter for predicting the decay process duration. This parameter known as “half-life” can be applied to determine an “epidemic regression half-life” that represents the time required to reduce the daily number of SARS-CoV-2-positive cases by 50%. At each half-life, 50% of the remaining daily contaminations are eliminated. Thus, by accumulating percentages of regression after each half-life, 99.2% of the epidemic should have disappeared after seven half-lives. As the number of half-lives increases, the objective of eliminating 100% of the contaminations becomes closer. After 10 half-lives, the percentage of elimination will be > 99.9%. This apparent requirement at the level of the last decimals is important since a few dozen residual infections can relaunch the epidemic. The “zero-COVID-19” strategy applied in several Southeast Asian countries, which implemented very strict containment measures, is based on this rationale. In New Zealand, which benefited from its insular situation and reached the zero-COVID-19 objective at the end of the first lockdown, we calculated an epidemic half-life of 4.2 days and an epidemic regression duration required to reach zero-COVID-19 starting from the date of lockdown set-up of 60 days, i.e. equaling 14 half-lives [6]. We concluded that ~ 14 times the epidemic half-life would be the requested time to get closer to the 100% elimination of viral circulation from the population.

**Fig. 1:**
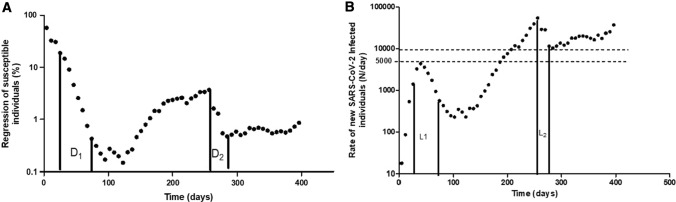
Usefulness of the epidemiokinetic tools. Regression of SARS-CoV-2-susceptible individuals (**A**) and rate of new SARS-CoV-2-infected individuals (**B**) in France from February 02, 2020 to March 29, 2021 represented in a semi-logarithmic scale. The two decay periods (D1 and D2) showing regression half-lives of ~ 10 days correspond to the two lockdowns (L1, 16 March-11 May 2020 and L2, 02-28 November 2020). Our data show how the proportion of the susceptible population changes with time and rapidly decreased over the lockdown, clearly demonstrating its effectiveness in controlling epidemic, although insufficiently to reach the Zero-COVID state

Therefore, our epidemiokinetic tools appear useful for calculating the optimal lockdown length to reach any desired objective in viral elimination. Following the first wave, lockdown in New Zealand lasted 33 days, i.e. ~ 8 times the epidemic half-life. This period of confinement resulted in > 99.6% of epidemic decay. The momentum of this lockdown then continued for a few weeks, leading close to the zero-COVID-19 aim. In France, the first confinement lasted 55 days, i.e. ~ 5.5 times the measured half-life of 10 days (Fig. 1A), allowing an epidemic regression of ~ 97.7%, which was insufficient to reach the zero-COVID-19 since hundreds of daily-contaminated cases persisted and were not optimally isolated, resulting in SARS-CoV-2 spread persistence. The second lockdown decided on October 29, 2020 lasted 4 weeks before shops reopened on November 28, i.e. only 3 times the calculated 10-day epidemic half-life. At this step, only 87.5% of the epidemic regression process was reached with ~ 10,000 residual daily contaminations. Thereafter, resumption of “normal” life stopped the epidemic decay and led to the maintenance of infections on a relatively high plateau that finally resulted in a frightening exponential rate increase since mid-March, due to the UK SARS-CoV-2-variant prevalence increase to above 80%.

## Zero-COVID

Our epidemiokinetic tools underscore the importance of maintaining restrictions over time after a short-term confinement to allow moving toward zero-COVID-19. Considering the incidence of 40,000 new infections/day in France (end of March 2021) and taking into account the latency time required to observe lockdown-attributed effects on infection (8–15 days), the incidence of new infections after a 30-day lockdown would best be around 10,000 cases/day. This value was reached after the second 4-week lockdown in October/November 2020 that led to a 2-month prolonged plateau of infections before further acceleration, which justified regional lockdowns in the departments with the highest infection and hospital saturation rates followed by a nationwide lockdown, which started on April 05, 2021 and ended recently on May 03, 2021.

It is possible using the epidemiokinetic tools to estimate the time required for an effective lockdown on a factual basis. Observers stated that a strict 3-week confinement might allow reopening of social activities, such as restaurants, sports, cultural, and leisure life. In our opinion, a strict lockdown of insufficient duration will require a follow-up “stop and go” period and never allow reaching zero-COVID-19. To achieve this objective, the population would have to be confined for a period of at least eight half-lives, i.e. almost 3 months in France if restrictions decided by health authorities allow for a 10-day half-life, as calculated during the two confinement-periods [6].

Are the European countries ready to confine their population for 3 months to reach zero-COVID-19? An alternative strategy to zero-COVID-19 would be to define the adequate threshold of daily SARS-CoV-2-positive case rate to be achieved with strict containment and then maintain that threshold using less stringent measures. The time to reach the targeted threshold could be translated into a number of epidemic half-lives that must be achieved before reducing the restrictions. One pertinent objective would be to go below a threshold allowing applying an effective strategy of testing, contact tracing and self-isolation, i.e. a threshold of 5000/day contaminations in France as acknowledged by health authorities. By remaining on the decay dynamics obtained during the second lockdown in November, this threshold could have been reached with an additional 10-day confinement. However, with the likely early stopping of restrictions, decline in decay had abruptly stopped at ~ 10,000/day contaminations around December 1, before increasing in mid-march 2021 as a third wave (Fig. 1B).

Following a period of restrictions, mandatory in almost all European countries, a too brutal lockdown release may lead to the epidemic resumption. Policy-makers and observers should not sell illusions that may affect the resilience displayed by citizens. Disappointment could be worse for mental health. Decision-making must be timely. The faster the restraint measures are decided, the more effective they will be as in countries that have achieved zero-COVID-19. Although its short-term benefits will not be sufficient to prevent hospital saturation and limit restrictions, mass vaccination represents the only way to exit from this slump situation, by protecting people at risk of developing severe COVID-19 and thereafter increasing herd immunity to limit the viral spread.

## Conclusion

Understanding the exact scope of each SARS-CoV-2 epidemic KPI is essential to evaluate the interventions undertaken by policymakers and enhance the population adherence. Our inter-disciplinary approach provides epidemiokinetic tools that may help in monitoring lockdown efficacy and assessing its adequate duration.
